# Antibodies to Highly Pathogenic A/H5Nx (Clade 2.3.4.4) Influenza Viruses in the Sera of Vietnamese Residents

**DOI:** 10.3390/pathogens10040394

**Published:** 2021-03-25

**Authors:** Tatyana Ilyicheva, Vasily Marchenko, Olga Pyankova, Anastasia Moiseeva, Tran Thi Nhai, Bui Thi Lan Anh, Trinh Khac Sau, Andrey Kuznetsov, Alexander Ryzhikov, Rinat Maksyutov

**Affiliations:** 1 State Research Center of Virology and Biotechnology “Vector”, Rospotrebnadzor, Koltsovo, Novosibirsk 630559, Russia; marchenko_vyu@vector.nsc.ru (V.M.); pyankova_og@vector.nsc.ru (O.P.); chalaya_aa@vector.nsc.ru (A.M.); ryzhik@vector.nsc.ru (A.R.); maksyutov_ra@vector.nsc.ru (R.M.); 2 Department Natural Sciences, Novosibirsk State University, Novosibirsk 630090, Russia; 3 Russian-Vietnamese Tropical Research and Technology Centre, Hanoi 650000, Vietnam; tbnnhai@yahoo.com (T.T.N.); lananhrus@yahoo.com (B.T.L.A.); sau_tk@yahoo.com (T.K.S.); tropcenterhanoi@mail.ru (A.K.)

**Keywords:** highly pathogenic avian influenza virus, H5N6 (clade 2.3.4.4), human sera

## Abstract

To cause a pandemic, an influenza virus has to overcome two main barriers. First, the virus has to be antigenically new to humans. Second, the virus has to be directly transmitted from humans to humans. Thus, if the avian influenza virus is able to pass the second barrier, it could cause a pandemic, since there is no immunity to avian influenza in the human population. To determine whether the adaptation process is ongoing, analyses of human sera could be conducted in populations inhabiting regions where pandemic virus variant emergence is highly possible. This study aimed to analyze the sera of Vietnamese residents using hemagglutinin inhibition reaction (HI) and microneutralization (MN) with A/H5Nx (clade 2.3.4.4) influenza viruses isolated in Vietnam and the Russian Federation in 2017–2018. In this study, we used sera from 295 residents of the Socialist Republic of Vietnam collected from three groups: 52 samples were collected from households in Nam Dinh province, where poultry deaths have been reported (2017); 96 (2017) and 147 (2018) samples were collected from patients with somatic but not infectious diseases in Hanoi. In all, 65 serum samples were positive for HI, at least to one H5 virus used in the study. In MN, 47 serum samples neutralizing one or two viruses at dilutions of 1/40 or higher were identified. We postulate that the rapidly evolving A/H5Nx (clade 2.3.4.4) influenza virus is possibly gradually adapting to the human host, insofar as healthy individuals have antibodies to a wide spectrum of variants of that subtype.

## 1. Introduction

Humanity has acquired knowledge to control most anthroponotic infections, such as measles, poliomyelitis, smallpox, and mumps. However, recently, problems related to zoonotic human infections have emerged. Since the natural reservoirs of these pathogens are unlimited, they are difficult or often impossible to control.

Influenza A virus belongs to the genus *Alphainfluenzaviruses* of the *Orthomyxoviridae* family and has a segmented genome consisting of single-stranded RNA segments of negative polarity [1]. Influenza A viruses are divided into subtypes (serotypes) based on the genetic and antigenic characteristics of its two surface glycoproteins, hemagglutinin (HA) and neuraminidase (NA). The nomenclature of these viruses is based on a combination of the HA (H1–H18) and NA (N1–N11) subtypes. Wild waterfowl are a natural reservoir for all influenza A subtypes [2], except for H17N10 and H18N11, which were recently found in bats [3,4]. Influenza A viruses can also be detected in a wide variety of hosts including humans, swine, horses, dogs, cats, and sea mammals.

Pandemic influenza A virus appears in the human population every 10–30 years. There is currently no immunity to these viruses; therefore, the resultant pandemics cause high morbidity and often high mortality. It is supposed that pandemic influenza A viruses emerge because of the reassortment of viruses in humans and animals or the adaptation of zoonotic viruses to humans [5].

Reassortment occurs when two strains of influenza A virus coinfect one cell and can cause procreation of the new reassortant virus, which contains a new set of genes [6]. The influenza pandemics in 1957 and 1968 were caused by reassortant viruses containing genes of influenza A viruses of humans and birds [7]. The first pandemic of the 21st century was caused by a virus that emerged after multiple reassortments of human-, avian-, and swine-origin influenza A viruses [8].

The views on the cause of the 1918 pandemic (the so-called “Spanish flu”) differ among experts. Some support the idea that the virus was directly introduced into the human population (without reassortment), while others believe that the pandemic virus emerged after multiple genome reassortments of avian and mammalian, and possibly swine and/or human, viruses that had emerged during the years preceding the pandemic of 1918 [9].

Regardless of the exact mechanism of the emergence of a new virus variant, there may be a certain period before the pandemic begins that is needed by the virus for optimal adaptation to the human host [10].

If this assumption is correct, then an understanding of whether the adaptation process is ongoing is possible by analyzing antibody levels in the sera of human populations inhabiting regions where the emergence of pandemic virus variants is most likely.

The objective of this research was to analyze the sera of Vietnamese residents using the hemagglutinin inhibition (HI) test and virus microneutralization (MN) with avian influenza viruses isolated in Vietnam and the Russian Federation in 2017–2018.

## 2. Results

**Figure 1:**
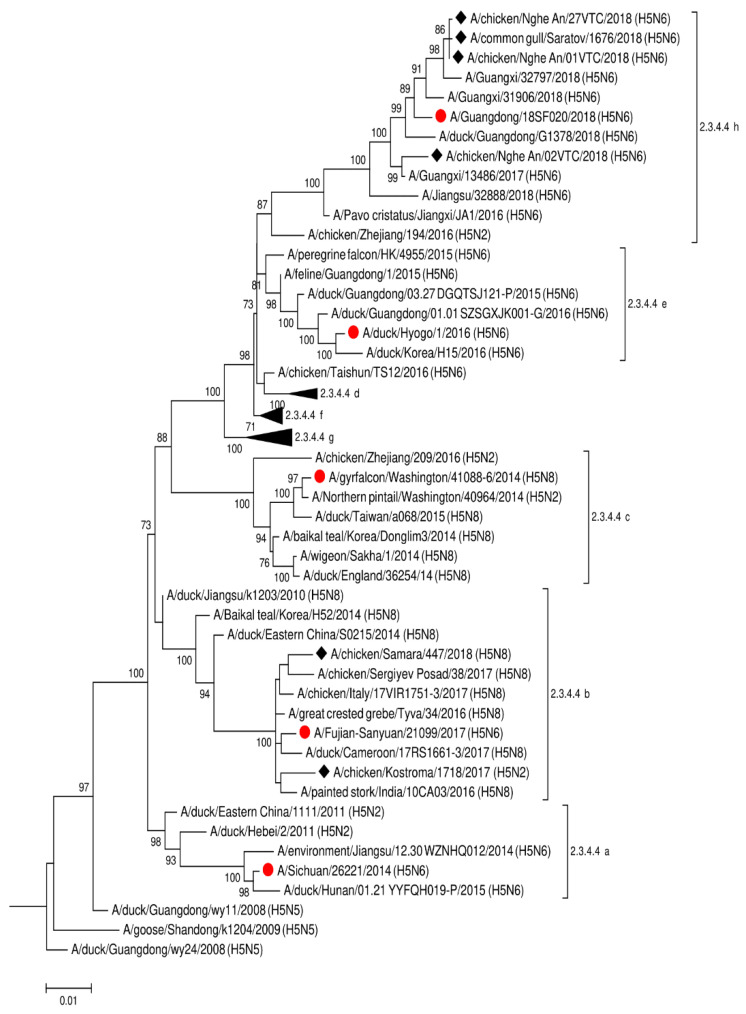
The phylogenetic tree for HA of A(H5Nx) influenza viruses. Viruses used in this study are indicated by rhombs. Candidate vaccine viruses are indicated by circles (according to WHO recommendations https://www.who.int/influenza/vaccines/virus/characteristics_virus_vaccines/en/, accessed on 5 March 2021).

For sera analyses, we selected influenza A virus strains that were isolated from poultry in Vietnam in 2018 and poultry and wild birds in Russia in 2017–2018. These strains were selected based on a phylogenetic analysis, which revealed a high degree of identity between the strains isolated in Russia and Vietnam (Figure 1). It was determined that the A/chicken/Nghe An/27VTC/2018 (H5N6), A/chicken/Nghe An/01VTC/2018 (H5N6), and A/common gull/Saratov/1676/2018 (H5N6) strains belonged to the genetic clade 2.3.4.4h. At that time, the strain A/chicken/Kostroma/1718/2017 (H5N2) was selected for comparison as a representative of clade 2.3.4.4b, which circulated widely in Eurasia. The study of antigenic properties is consistent with the phylogenetic analysis. Data are presented in Table 1.

As shown in Table 1, the A/chicken/NgheAn/27VTC/2018 (H5N6) and A/chicken/NgheAn/01VTC/2018 (H5N6) viruses cross-reacted with sera against A/common gull/Saratov/1676/2018 (H5N6) as effectively as the homological virus (the reciprocal of the titer in the HI test is 320). Vietnamese strains did not react with other ferret reference sera obtained against the A/H5N6 viruses isolated in Russia.

For the analysis of sera against influenza virus subtype A/H5, we selected the following viruses: low pathogenic A/chicken/Kostroma/1718/2017 (H5N2) virus (LPAI) and highly pathogenic A/common gull/Saratov/1676/2018, A/chicken /Nghe An/01VTC/2018, and A/chicken/Nghe An/27VTC/2018 (H5N6) viruses (HPAI).

The sera were tested using the HI test with human vaccine viruses, highly and lowly pathogenic viruses of the A/H5 subtype, highly pathogenic A/H7N9 virus, and lowly pathogenic A/H9N2 virus.

Testing with vaccine strains showed that antibodies with significant titers (40 and higher) to the A/Michigan/45/2015 (H1N1pdm09) virus were detected in 29 samples (14, 2, and 13 from three groups, respectively). Antibodies to the A/Singapore/INFIMH-16-0019/2016 (H3N2) vaccine virus were detected in 55 samples (14, 6, and 35 from each group, respectively) (data not shown).

None of the tested serum samples reacted in HI or MN with A/Anhui/1/2013 (H7N9) virus at dilutions of 1:40 or higher. Four samples had a titer of 20 in the HI test, but not in MN. Fifty-seven samples reacted with the A/chicken/Primorsky Krai/03/2018 (H9N2) virus in the HI test, of which 0, 1, and 56 samples were from the three groups, respectively (data not shown).

The results of the sera analyses in HI and MN for A/H5Nx (2.3.4.4.) avian influenza viruses are presented in Table 2. Data are presented only for samples that were positive in at least one study.

As shown in Table 2, only one serum from the first group reacted in the HI test with one of the viruses, A/chicken/Kostroma/1718/2017 (H5N2) 2.3.4.4. Seven serum samples from the second group reacted in the HI test with one or two A/H5 viruses. The largest number of positive serum samples was in the third group: 55 samples reacted in the HI test and two samples in MN with the A/chicken/NgheAn/01VTC/2018 (H5N6) virus, 59 serum samples were positive in the HI test and only one in MN with A/chicken/Kostroma/1718/2017 (H5N2) virus, 51 serum samples in HI and 47 in MN with A/chicken/NgheAn/27VTC/2018 (H5N6) virus, and 54 serum samples were positive in HI and 27 in MN with the A/common gull/Saratov/1676/2018 (H5N6) virus.

## 3. Discussion

The first documented outbreak of human infection with the avian influenza A/H5 virus occurred in Hong Kong in 1997. Since then, A(H5N1) has caused diseases in 861 people, and 455 cases were fatal (data up to 20 September 2020). In Vietnam, 127 confirmed cases of human infection with the A(H5N1) virus were detected between 2003 and 2014, 64 of which were fatal. No human cases of highly pathogenic avian influenza A/H5 have been reported in Vietnam since 2015 [11]. However, outbreaks in poultry and wild birds have been reported in Vietnam to date, including those caused by the most rapidly evolving H5N6 subtype [12].

Today, HPAI H5N6 is one of the few subtypes of the avian influenza virus that can infect humans [13]. Studies have shown that the H5N6 virus originated from a common precursor strain of the clade 2.3.4.4 subtype H5 as a result of reassortment with the A/duck/Guangxi/2281/2007 (H6N6) strain [13,14,15,16]. However, there is evidence that the origin of the H5N6 viruses followed other evolutionary paths [17]. According to one of the hypotheses, this virus appeared in the period from 2010 to 2012 as a result of reassortment of the H5N2 virus of clade 2.3.4.4 with the A/duck/Guangxi/2281/2007 (H6N6) strain, followed by reassortment of the six internal genes with the H5N1 influenza virus of clade 2.3.2.1c isolated from chickens [14]. The H5N6 virus, the so-called “reassortant A”, which developed along this pathway, circulates in Xinjiang, Jilin, and Northern China [14,15,18]. In 2013, the virus spread to Western China, where it caused outbreaks in Sichuan, Vietnam, and Laos [14,19].

Another variant of the H5N6 influenza virus, named “reassortant B,” appeared in 2013 because of reassortment of H6N6 viruses with H5N8 viruses in clade 2.3.4.4 and subsequent resorting of genes with H5N1 viruses in clade 2.3.2.1c [14]. This virus also circulated in China, Vietnam, and Laos. Two years later, reassortant B underwent repeated reassortment with an influenza virus of the H9N2 subtype, resulting in a new variant of the H5N6 virus, reassortant C. The circulation of this variant was reported in the Yunnan and Guangdong provinces of China [17,18]. Regardless of the evolutionary way in which these reassortants emerged, it is known that all of them caused infectious diseases among humans [14,15,16]. Since 2014, H5N6 viruses (2.3.4.4) have caused 27 human infections, resulting in 15 deaths in China [20]. Most human cases are associated with A or B reassortants.

In 2014, H5N6 spread across Laos and Vietnam, resulting in huge economic losses due to outbreaks in poultry [18,21]. In 2016, H5N6 caused several outbreaks in Japan, Myanmar, and the Republic of Korea [14,15,21]. In 2017, outbreaks of H5N6 were reported in Taiwan and the Philippines. In addition, with wild migratory birds, HPAI H5N6 was introduced into Europe during this time. Outbreaks were reported in Greece, Germany, the Netherlands, and Switzerland [14,15,16,17,22]. To date, H5N6 circulation is limited to Asia and Europe.

In addition to influenza viruses of the A/H5 subtype, the H6, H7, H9, and H10 viruses also have pandemic potential, since there is confirmed evidence that they can cross the interspecies barrier and infect humans [23]. For example, highly pathogenic A(H7N9) viruses caused 1568 confirmed human cases, 616 of which were fatal (data up to 20 September 2020) [24].

To cause a pandemic, the virus has to overcome two main obstacles. First, the pathogen must be antigenically new to humans to ensure that herd immunity does not impede the rapid spread of the virus. Second, the virus must be effectively transmitted from person to person [25]. Thus, if the avian influenza virus is able to pass the second barrier, it could cause a pandemic, since there is no immunity to avian influenza virus in the human population.

Vietnam is considered one of the hotspots for the emergence of influenza viruses with epidemic and pandemic potential [26]. A/H5 highly pathogenic avian influenza viruses have been endemic in Vietnamese poultry for over a decade and a half. It has been shown that in the northern provinces of Vietnam, more than 30% of poultry have antibodies to the influenza A/H5 virus [26]. The Global Influenza Surveillance and Response System (GISRS) tracks the emergence of viruses with pandemic potential. Within the framework of GISRS, this work is being carried out at the WHO Reference Laboratory for H5 on the basis of the State Research Center of VB “Vector” of Rospotrebnadzor [27] and in the Socialist Republic of Vietnam [28,29,30].

However, during the adaptation process, virus variants may not cause clinical manifestations and may remain unnoticed during monitoring activities. It is even more difficult to detect the adaptation of lowly pathogenic avian influenza viruses to humans. In such cases, adaptation can be assessed by analyzing sera collected from residents of areas with a high risk of avian influenza.

In the present study, we investigated the sera of Vietnamese residents using MN and HI with highly pathogenic, lowly pathogenic, and vaccine influenza A viruses H5N2, H5N6, H7N9, H9N2, A/H1N1pdm09, and A/H3N2 subtypes. The analyses showed that only 10% of sera had significant titers in the HI test with the A/H1N1pdm09 vaccine virus and close to 20% with the A/H3N2 vaccine virus. This indicates a low level of population immunity against seasonal influenza, which represents a serious risk, since the probability of human infection with a seasonal and highly pathogenic influenza virus is increasing, and this event could lead to the emergence of a reassortant virus with new antigenic features and the ability to be transmitted from person to person.

In the tested sera, we did not find significant titers of antibodies against the A/Anhui/1/2013 (H7N9) virus; only four serum samples out of 295 had a titer of 1:20 in HI, but not in MN. With the A/chicken/PrimorskyKrai/03/2018 (H9N2) virus, 57 serum samples were positive. This is consistent with previous data on the wide circulation of the A(H9N2) virus in Vietnam, especially in live poultry markets [31], and relatively high antibody levels to this virus serotype in human sera [28]. Current concerns about H9N2 viruses are related to their ability to reassort with other avian influenza viruses, resulting in highly and lowly pathogenic viruses that can cross species barriers and infect humans [32].

Among all investigated viruses of the A/H5 subtype, only two viruses, A/chicken/Nghe An/27VTC/2018 (H5N6) 2.3.4.4 and A/common gull/Saratov/1676/2018 (H5N6) 2.3.4.4, had a significant number of positive serum samples in HI and MN. For other viruses, we found mostly anti-hemagglutination antibodies, but not neutralizing virus in cell cultures. However, the absence of neutralizing antibodies does not mean that humans have not been infected. Thus, Li et al. showed that after mice were infected with wildtype A/H7N9 virus, all animals after 14 days had high titers of HI antibodies in their sera, but not virus-neutralizing antibodies. At the same time, the recombinant virus, which contained genes for internal proteins from the PR8 strain, and the HA and NA genes from A/H7N9, induced significantly higher antibody levels in sera, detected in both HI and MN. The authors concluded that internal proteins of the A/H7N9 virus can influence the humoral immune response of the host [33].

We analyzed all sera in the HI test with horse, goose, and turkey red blood cells and demonstrated the highest titers with horse red blood cells, in accordance with [34]. It is not clear why, in the present study, many serum samples from the third group reacted with different A/H5 viruses in HI (but not in MN). Thus, 45 serum samples were positive in the HI test and all were included in the study of A/H5 viruses. At the same time, these viruses in most cases did not react in HI with heterologous ferret reference sera. However, antibodies may appear because of human infection with various A/H5 pathogens. However, in our opinion, it is highly probable that the presence of antibodies to different A/H5 viruses in the same serum samples can be explained by the fact that A/H5 viruses induce in humans (but not in ferrets) a wide range of anti-H5 cross-reactive antibodies. This may be because of frequent human contact with various virus subtypes, while, to obtain a reference serum, animals that do not have antibodies to any influenza virus are selected.

To understand this phenomenon and, more importantly, the process of avian influenza virus adaptation to humans, it is necessary to continue studies of the circulation of viruses in domestic and wild birds, as well as the sera of people living in “hotspot” regions of pandemic potential virus emergence. From the results of the present research, we can postulate that the rapidly evolving A/H5Nx influenza viruses (clade 2.3.4.4) are gradually adapting to human hosts, in so far as healthy individuals have neutralizing and anti-hemagglutinating antibodies to a wide range of viruses of this subtype. The number and profiles of serum samples tested in this work were limited, so our results may be misleading; thus, we cannot say with confidence that the process of adaptation of H5 avian viruses to humans is underway. Nevertheless, our results allow us to make such an assumption.

It should be noted that in the human population, there are diseases that are caused by pathogens that previously circulated only among animals: SARS, the Middle East respiratory syndrome, coronavirus disease 2019 (COVID-19), acquired immune deficiency syndrome, and pandemics caused by Chikungunya and Zika viruses [35]. It is believed that, in the future, new viruses that are pathogenic to humans will emerge, owing to the presence of an unlimited natural reservoir of animal viruses. One of the most dangerous among them includes highly pathogenic influenza viruses. It is possible that pandemics similar to the Spanish flu will emerge [36]. Therefore, it is important to conduct comprehensive surveillance of avian and mammalian influenza viruses, including monitoring human sera for the presence of antibodies to animal influenza viruses.

## 4. Materials and Methods

**Sera**. The blood serum research was approved by the Ethics Committee IRB 00001360, affiliated with SRC VB Vector (No.2 d.d. Protocol, May 2008). The study used blood sera collected from residents of the Socialist Republic of Vietnam; 52 samples (No 1-52) were collected from private households in Nam Dinh province, where poultry deaths have been reported (2017) [37]; 96 samples (No 53-148) were collected in Hanoi from healthy donors (2017), and 147 samples (No 200-346) were collected from patients with non-communicable diseases in hospitals in Hanoi (2018). Blood samples were collected, on condition of anonymity, from individuals of different age groups: 18–55 years (90%), 56–64 years (8%) and 65 years and older (2%).

Before HI testing, sera were treated with receptor-destroying enzyme and hemadsorbed on horse RBCs, before MN sera were heat-inactivated for 30 min at 56 °C, as described by [34,38].

**Viruses**. A/Michigan/45/2015 (H1N1) pdm09 and A/Singapore/INFIMH-16-0019/2016 (H3N2) vaccine influenza viruses were kindly provided by the WHO Collaborating Center in Atlanta, United States. The WHO Collaborating Center in Beijing, China, kindly provided the A/Anhui/01/2013 (H7N9) virus. Virus A/chicken/Kostroma/1718/2017 (H5N2) [39], virus A/common gull/Saratov/1676/2018 (H5N6) [40,41], A/chicken/PrimorskyKrai/03/2018 (H9N2), A/chicken/NgheAn/01VTC/2018 (H5N6), and A /chicken/NgheAn/27VTC/2018 (H5N6) were isolated by the authors. A maximum-likelihood tree based on the Hasegawa–Kishino–Yano model was built using MEGA 6.06 software (http://www.megasoftware.net/, accessed on 5 March 2021) with 1000 bootstrap replicates.

**HI test and MN**. The hemagglutination inhibition (HI) test and microneutralization (MN) method were performed as described by [38,41]. Horse, goose, and turkey red blood cells were used in HI tests. Highest titers were obtained with horse red blood cells. Each serum was tested three times in the HI test with horse red blood cells, and the results differed by no more than 2 times. A lower value was taken for the serum titer. Sera from recovered ferrets infected with analyzed virus strain were used as a positive control. Negative control was represented by sera of non-immune ferrets and human sera without antibodies to avian influenza virus.

## Funding

The study was conducted at the expense of targeted subsidies by order of the Government of the Russian Federation of July 13, 2019 No. 1536-r. and by State Assignment no. 1/21 and no. 3/21 (SRC VB “Vector”).

## Figures and Tables

**Table 1:** HI test of H5Nx clade 2.3.4.4 viruses with ferret reference antisera and horse red blood cells.

Virus	Subtype	Clade	Reverse Titer with Antisera									
			A/duck/England/36254/ 2014	A/Northern Pintail/WA/40964/2014	A/Sichuan/26221/2014 RG42A	A/gyrfalcon/WA/41088/2014 RG43A	A/great crested grebe/Tyva/34/2016	A/wigeon/Sakha/1/2014	A/chicken/SergiyevPosad/ 38/2017	A/chicken/Kostroma/1718/ 2017	A/common gull/Saratov/1676/2018	A/chicken/Nghe An/27VTC/2018
A/duck/England/36254/2014	H5N8	2.3.4.4c	**640**	5120	5120	640	1280	1280	1280	2560	NA	NA
A/Northern Pintail/WA/40964/2014	H5N2	2.3.4.4c	320	**5120**	2560	2560	640	640	160	1280	<20	<20
A/Sichuan/26221/2014 RG42A	H5N6	2.3.4.4a	640	5120	**5120**	320	640	320	320	1280	<20	NA
A/gyrfalcon/WA/41088/2014 RG43A	H5N8	2.3.4.4c	640	5120	1280	**5120**	1280	1280	320	1280	<20	<20
A/great crested grebe/Tyva/34/2016	H5N8	2.3.4.4b	320	5120	2560	2560	**1280**	640	640	1280	<20	<20
A/wigeon/Sakha/1/2014	H5N8	2.3.4.4c	640	10,240	2560	5120	640	**640**	640	2560	<20	<20
A/chicken/SergiyevPosad/38/2017	H5N8	2.3.4.4b	160	2560	2560	2560	320	640	**160**	1280	<20	<20
A/chicken/Kostroma/1718/2017	H5N2	2.3.4.4b	320	10,240	2560	2560	640	640	320	**5120**	<20	<20
A/common gull/Saratov/1676/2018	H5N6	2.3.4.4h	<20	<20	80	<20	<20	<20	<20	<20	**320**	80
A/chicken/NgheAn/27VTC/2018	H5N6	2.3.4.4h	<20	320	40	20	<20	<20	<20	<20	320	**80**
A/chicken/NgheAn/01VTC/2018	H5N6	2.3.4.4h	NA	640	80	<20	<20	<20	<20	<20	320	160
A/chicken/Vietnam/NCVD-15A59/2015	H5N6	2.3.4.4f	320	10,240	2560	2560	640	640	160	640	40	<20

HI titers of sera with a homologous viruses are highlighted in bold.

**Table 2:** MN and HI test of human sera with H5 avian influenza viruses.

Human Serum Sample	Group	A/chicken/ NgheAn/01VTC/ 2018 (H5N6) 2.3.4.4		A/chicken/ Kostroma/ 1718/ 2017 (H5N2) 2.3.4.4		A/chicken/ NgheAn/27VTC/2018 (H5N6) 2.3.4.4		A/common Gull/Saratov/1676/2018 (H5N6) 2.3.4.4	
		HI	MN	HI	MN	HI	MN	HI	MN
20	1			≥160					
56	2			40					
60	2	40							
82	2			40					
100	2	40		40					
105	2	80							
128	2	40		40					
134	2			40					
200	3	≥160		≥160		≥160	160	160	80
203	3	80	80	160		160	80	≥160	80
209	3	≥160	80	160		≥160	160	≥160	160
212	3	≥160		160		80	80	≥160	160
213	3	≥160		160		160	80	≥160	80
214	3	≥160		160		160	80	≥160	80
221	3	80		≥160		80	80	≥160	160
222	3	40		160		40	80	80	80
231	3			40					
232	3			40					
235	3	80		160		160	320	≥160	80
237	3	80		≥160		160	320	≥160	160
239	3	80		160		40	80	160	160
240	3	≥160		≥160		160	80	≥160	320
241	3	≥160		160		160	80	≥160	320
250	3	80		≥160		80	80	160	160
251	3	40		160					
255	3	≥160		≥160		≥160	80	≥160	320
257	3	≥160		≥160		80	80	80	160
258	3	≥160		≥160		160	80	≥160	160
259	3	≥160		≥160		160	160	≥160	160
268	3	≥160		≥160		80	160	≥160	80
270	3	80		≥160		40	80	80	
271	3	≥160		≥160		80	160	≥160	80
272	3	≥160		≥160		80	160	≥160	
273	3	80		≥160		80	80	≥160	320
274	3	80		≥160		80	80	≥160	80
275	3	≥160		≥160		≥160	160	≥160	
277	3	40		40					
278	3	≥160		160		80	160	≥160	80
281	3	≥160		≥160		80	160	160	
283	3	≥160		80		80	80	≥160	
284	3	≥160		≥160		≥160	160	≥160	
285	3			≥160		≥160	320	≥160	
286	3	≥160		≥160		80	160	≥160	
287	3	≥160		80		80	80	≥160	
288	3			80		40		≥160	
289	3	≥160		≥160		160	160	≥160	
290	3	80		80		80	80	80	
291	3	80		160		160	80	≥160	
292	3	80		≥160		160	80	≥160	
293	3	80		160		80	80	≥160	
294	3	80		≥160		80	80	≥160	
295	3	80		160		80	80	≥160	
296	3	80		160		40		40	
297	3	40		40				40	
298	3	40		80					
299	3	80		80		80	160	80	
300	3	80		80		80	160	80	
303	3	40		80		40		80	
304	3	40		80				160	
307	3	40		80				40	160
308	3	80		80		80	80	80	160
309	3	40		≥160		40	80	40	
310	3	40		80		40		40	
334	3	80		80	80	80	160	40	
335	3	80		80		80	160	80	80
338	3	80		80		80	160	40	80
339	3	80		80		80	160	80	
Total number of positive sera		59	2	65	1	51	47	54	27

Reciprocal serum titers are shown for the selected sera positive in HI and MN simultaneously at least for one virus. The positive serum in MN ≥ 40, the positive serum in HI test ≥ 40. Each serum was tested three times in HI test with horse red blood cells; the results differed by no more than 2 times, and a lower value was taken for the serum titer. Sera from recovered ferrets infected with analyzed virus strain were used as a positive control. Negative control was represented by sera of non-immune ferrets and human sera without antibodies to avian influenza virus. Negative control titer in all tests > 20, positive control titer from 320 to 640.

## Data Availability

Data sharing is not applicable to this article.
